# Unusual Nasopharyngeal Localization of Chronic Lymphocytic Leukemia: A Case Report

**DOI:** 10.7759/cureus.104691

**Published:** 2026-03-04

**Authors:** Monsif FADI, Said Anajar, Hafsa Chahdi, Fatima Azzahra Lahlou, Nouama Bouanani

**Affiliations:** 1 Oncopathology, Cancer Biology and Environment Laboratory, Center for Doctoral Studies (CEDOC) Faculty of Medicine, Mohammed VI University of Health Sciences (UM6SS), Casablanca, MAR; 2 Otolaryngology - Head and Neck Surgery, Faculty of Medicine, Mohammed VI University of Health Sciences (UM6SS), Casablanca, MAR; 3 Pathology, Cheikh Khalifa International University Hospital, Mohammed VI University of Health Sciences (UM6SS), Casablanca, MAR; 4 Biochemistry, Health and Environment Laboratory, Ain Chock Faculty of Sciences, Hassan II University, Casablanca, MAR; 5 Biochemistry, Mohammed VI National Laboratory, Mohammed VI University of Health Sciences (UM6SS), Casablanca, MAR; 6 Hematology, Faculty of Medicine, Mohammed VI University of Health Sciences (UM6SS), Casablanca, MAR

**Keywords:** chronic lymphocytic leukemia (cll), ear nose throat (ent), hematology case report, nasopharynx cancer, traitement médical

## Abstract

Chronic lymphocytic leukemia (CLL) is a common B-cell malignancy that typically involves the peripheral blood, bone marrow, and lymphoid tissues, whereas extranodal involvement remains rare. Nasopharyngeal localization is exceptionally uncommon and may mimic primary head and neck tumors, leading to diagnostic challenges. We report the case of a 64-year-old man presenting with progressive left-sided nasal obstruction. Clinical examination revealed bilateral cervical lymphadenopathy and splenomegaly. Imaging studies demonstrated a well-defined mass occupying the posterosuperior wall of the nasopharynx, involving the Rosenmüller fossae and Eustachian tube orifices, without bone invasion. Histopathological examination showed a dense infiltration of small, monomorphic lymphocytes, and immunohistochemistry was positive for CD20, CD5, CD23, and Bcl-2. Peripheral blood lymphocytosis with a Matutes score of 5/5 confirmed the diagnosis of CLL, and cytogenetic analysis showed no deletion of 17p (p53). The patient was treated with six cycles of rituximab-bendamustine, resulting in complete regression of the nasopharyngeal mass and reduction in splenomegaly, with minimal residual lymphadenopathy and good clinical and biological tolerance. This case highlights the importance of considering CLL in the differential diagnosis of nasopharyngeal masses and emphasizes the role of combined imaging, histology, and immunophenotyping in establishing an accurate diagnosis and guiding appropriate management.

## Introduction

Chronic lymphocytic leukemia (CLL) is a hematologic malignancy characterized by the monoclonal accumulation of mature B lymphocytes that are morphologically normal but immunologically dysfunctional in the peripheral blood, bone marrow, lymph nodes, and other lymphoid organs [1]. It represents the most common form of leukemia in adults in Western countries and typically presents with persistent lymphocytosis, peripheral lymphadenopathy, and bone marrow infiltration [1]. Extranodal involvement remains uncommon, and nasopharyngeal localization, particularly in the cavum, is an exceptional presentation [1,2]. This atypical clinical presentation can easily be mistaken for primary head and neck tumors, especially undifferentiated nasopharyngeal carcinoma (UCNT), thereby complicating the initial diagnosis [3]. Accurate diagnosis, therefore, relies on a multimodal approach integrating imaging findings, histopathological examination, and immunophenotypic characterization [1].

Beyond the diagnostic challenge, the clinical and biological significance of extranodal nasopharyngeal involvement in CLL remains poorly defined. In particular, it is unclear whether such an unusual localization reflects an aggressive disease phenotype or merely an atypical pattern of dissemination in biologically indolent CLL.

The present case aims to contribute to this unresolved issue by describing a rare nasopharyngeal localization of CLL and by correlating its clinicopathological features, molecular profile, and therapeutic response, thereby providing insight into the biological heterogeneity of CLL.

## Case presentation

A 64-year-old male patient presented with a left-sided nasal obstruction that had been progressively worsening over several weeks. Otolaryngological examination revealed a tumorous process located in the nasopharynx, prompting further diagnostic investigations. Clinical examination also identified bilateral lateral cervical lymphadenopathy, with the largest lymph node measuring approximately 4 cm in diameter in the left submandibular region, associated with splenomegaly extending 3 cm below the left costal margin.

**Figure 1:**
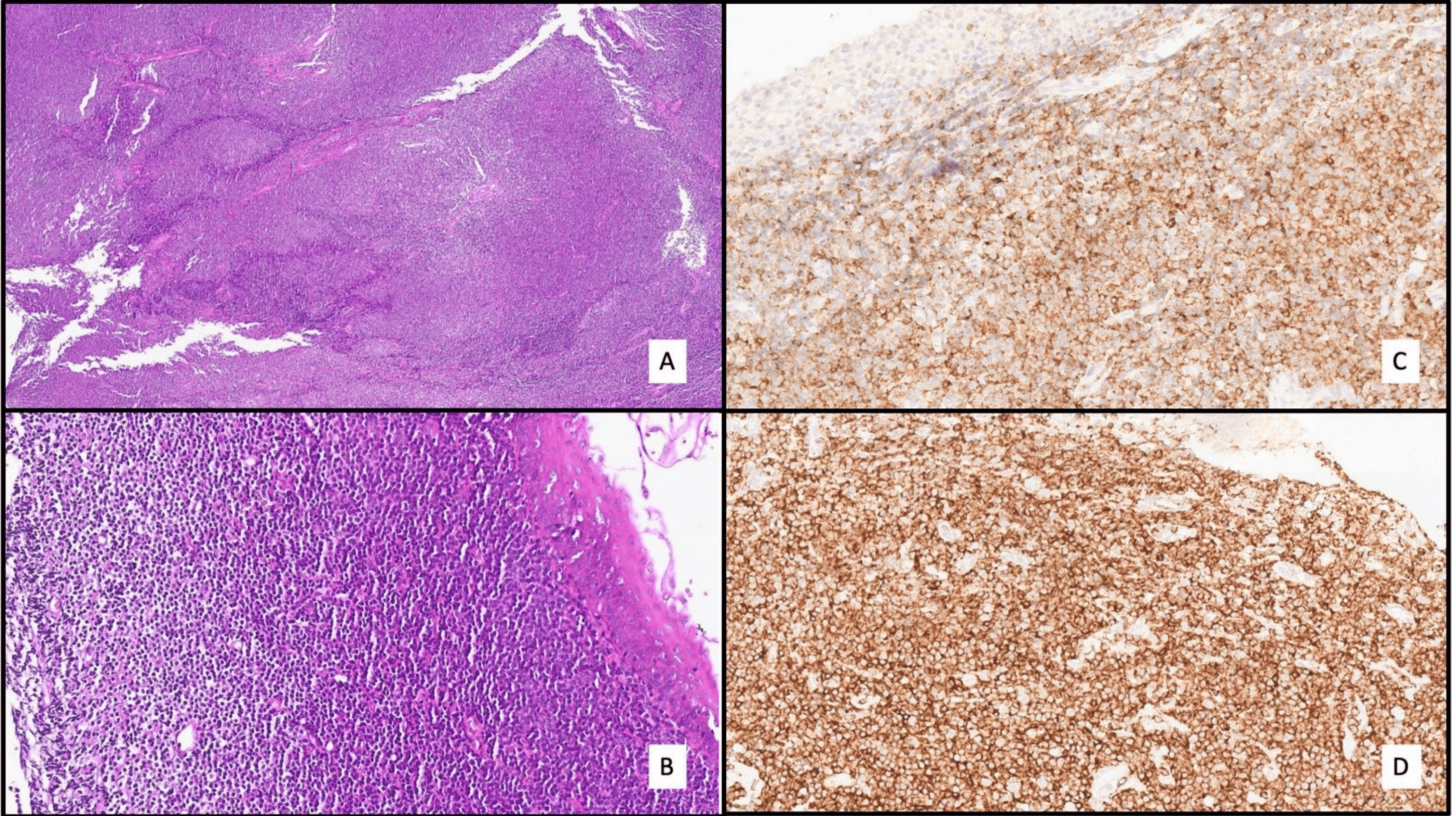
Lymphoid infiltration of chronic lymphocytic leukemia in the nasopharynx: histological and immunohistochemical study. (A) Histological section (hematoxylin and eosin, ×40) showing a diffuse lymphoid proliferation in the nasopharynx. (B) High-power view (hematoxylin and eosin, ×200) revealing a monomorphic infiltration of small lymphocytes with round nuclei and dense chromatin. (C) Immunohistochemistry for CD5 (×200): positive staining of the infiltrating lymphocytes. (D) Immunohistochemistry for CD20 (×200): diffuse and strong expression confirming the B-cell nature of the proliferation.

Histological examination of the nasopharyngeal biopsies showed a mucosa infiltrated by a dense lymphoid proliferation with a subtle follicular organization (hematoxylin and eosin, ×40). At higher magnification (hematoxylin and eosin, ×200), the proliferation was composed of small monomorphic lymphocytes with round to slightly irregular nuclei, dense chromatin, and no prominent nucleoli. Immunohistochemical analysis demonstrated a diffuse and strong expression of CD23 and an intense, homogeneous membranous expression of CD5, confirming the B-cell lymphoproliferative nature of the lesion and supporting the diagnosis of nasopharyngeal localization of CLL (Figure 1).

The complete blood count revealed leukocytosis with marked lymphocytosis, moderate thrombocytopenia, elevated lactate dehydrogenase (LDH) levels, and hypogammaglobulinemia (Table 1). Immunophenotyping demonstrated a Matutes score of 5/5, confirming the diagnosis of CLL. Histopathological examination of the nasopharyngeal mass revealed an immunohistochemical profile consistent with CLL (CD20+, CD5+, CD23+, BCL2+, CD10-, BCL6-, cyclin D1-, Ki-67: 15%). Fluorescence in situ hybridization (FISH) analysis did not detect high-risk cytogenetic abnormalities, including deletion of the TP53 locus at 17p13 [4]. Molecular analysis of the immunoglobulin heavy chain variable region (IGHV) gene was performed using polymerase chain reaction (PCR) amplification followed by sequencing [5]. The analysis revealed a mutated IGHV status, with more than 2% deviation from the closest germline sequence. This molecular profile is associated with a more favorable prognosis and is consistent with the good clinical response observed in this patient. Based on the presence of lymphadenopathy, splenomegaly, and thrombocytopenia, the disease was classified as Binet stage B. Altogether, these findings supported the diagnosis of CLL [6], with an unusual extranodal nasopharyngeal localization and features suggestive of a clinically significant disease burden.

**Table 1:** Laboratory findings at diagnosis. LDH, lactate dehydrogenase

Parameter	Patient value	Normal range
White blood cells (Elements/mm³)	18,810/mm³	4,000-10,000/mm³
Lymphocytes (Elements/mm³)	13,788/mm³	1,000-4,000/mm³
Hemoglobin (g/dL)	15.2 g/dL	13-17 g/dL
Platelets (Elements/mm³)	142,000/mm³	150,000-400,000/mm³
LDH ( U/L)	420 U/L	135-225 U/L
Gamma globulins (g/dL)	0.4 g/dL	0.7-1.6 g/dL

The Matutes immunophenotypic scoring system, the Binet staging system, IGHV mutational status assessment, and FISH analysis for TP53 deletion are internationally validated academic tools routinely used in clinical and research settings and do not require any specific licensing [5,7].

Facial computed tomography (CT) revealed a soft-tissue density lesion 48 Hounsfield units (HU) involving the posterosuperior wall of the nasopharynx, showing moderate enhancement after contrast administration (62 HU). The mass obliterated the Rosenmüller fossae and the opening of the Eustachian tube. It measured 33 mm in anteroposterior diameter, 42 mm transversely, and 40.7 mm in height, resulting in a reduction of the nasopharyngeal airway. Additionally, mucosal thickening was observed in the right sphenoidal sinus, as well as in the left maxillary and frontal sinuses and several left ethmoidal cells, consistent with sinusitis. The left mastoid air cells and middle ear cavity were opacified, findings consistent with serous otitis media secondary to Eustachian tube obstruction. No retroauricular inflammatory signs or anterior displacement of the auricle were observed, and there was no clinical evidence of acute otomastoiditis.

**Figure 2:**
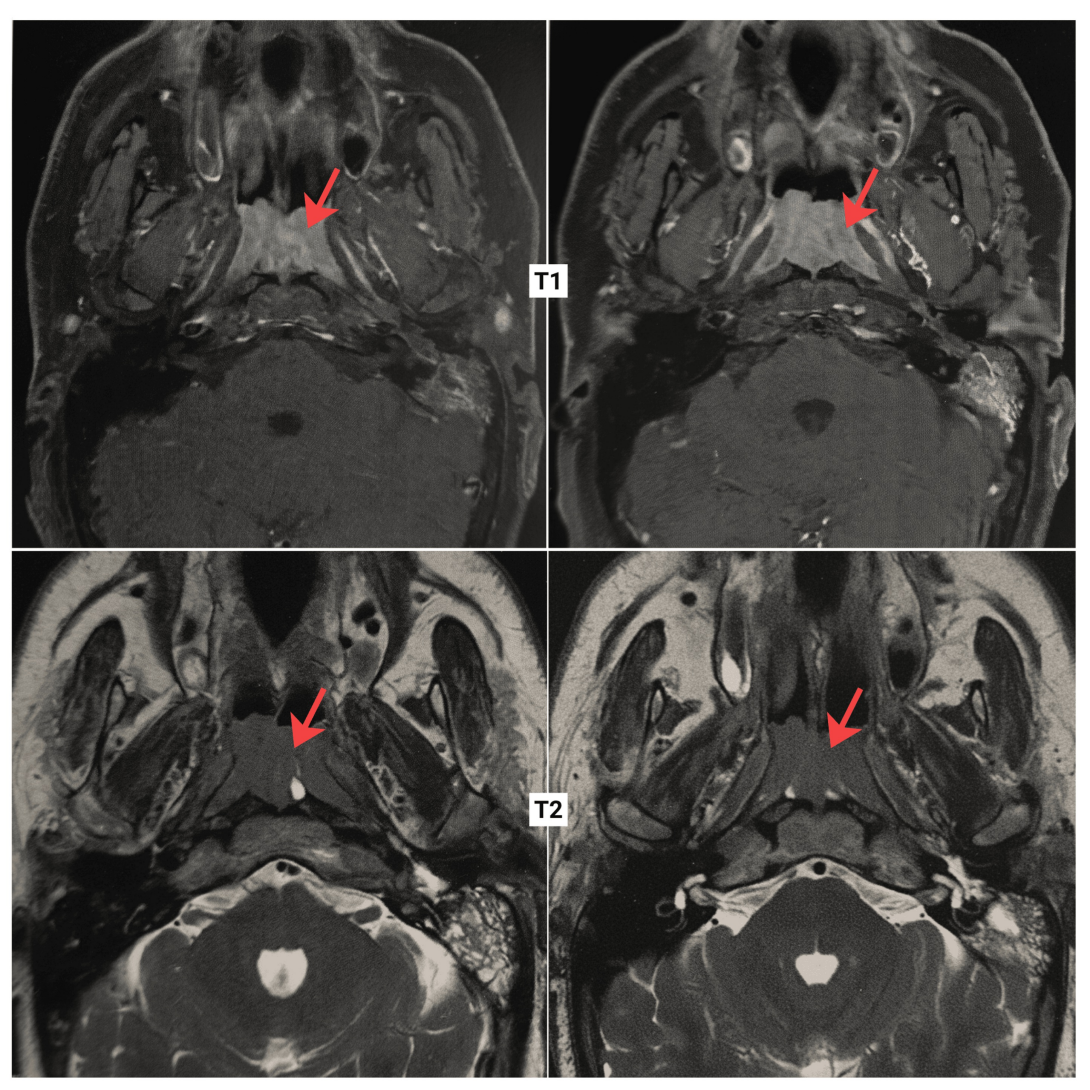
Axial T1- and T2-weighted MRI showing nasopharyngeal involvement in chronic lymphocytic leukemia. Topographic assessment showed that the mass was laterally limited by the nasopharyngeal wall without extension into the lateral pharyngeal spaces. Anteriorly, it reached the choanae, posteriorly it was in contact with the prevertebral muscles without significant invasion, superiorly it abutted the sphenoid sinus with no evidence of bony or intracranial extension, and inferiorly it extended to the pharyngeal and hypopharyngeal walls as a semi-circumferential mucosal thickening. Intense contrast enhancement was also observed at the tonsillar region, continuing with the mucosal thickening and extending to the lingual tonsils. The left mastoid cells and middle ear were opacified. Overall, the imaging findings were consistent with a localized nasopharyngeal lesion.

Magnetic resonance imaging (MRI) of the nasopharynx revealed a large nasopharyngeal mass occupying multiple recesses, including both Rosenmüller fossae and the Eustachian tube orifices. The lesion demonstrated intermediate signal intensity on T1 and T2 weighted images, with intense, homogeneous enhancement after gadolinium administration. The mass measured approximately 20 mm in maximum anteroposterior thickness, 40 mm in transverse diameter, and 42 mm in craniocaudal height (Figure 2).

The patient received six cycles of rituximab-bendamustine combination therapy. This regimen, well established in the management of CLL, combines the anti-CD20 monoclonal antibody rituximab, which enhances immune-mediated cytotoxicity against malignant B lymphocytes, with bendamustine, an alkylating agent with purine analog-like properties. After completion of six cycles, the patient demonstrated good clinical and biological tolerance, with no major treatment-related adverse events, allowing continued follow-up in an outpatient setting.

Post-treatment evaluation showed complete regression of the nasopharyngeal mass located on the posterosuperior wall of the nasopharynx. Residual lymphadenopathy was limited to small, millimetric cervical and thoraco-abdominal lymph nodes, without any lymph nodes of pathological size. Follow-up laboratory investigations revealed normalization of the white blood cell count and a significant decrease in peripheral lymphocytosis. Peripheral blood smear examination demonstrated the absence of circulating atypical lymphocytes, with no evidence of smudge cells or morphological signs of disease activity. Overall therapeutic assessment was consistent with a favorable hematological and radiological response. Additional imaging findings included a thin pericardial effusion, moderate prostatic hypertrophy, and degenerative disc disease, which were considered incidental and clinically stable.

## Discussion

CLL is the most common leukemia in adults in Western countries, accounting for approximately 25-30 % of all leukemias [4]. It is characterized by a monoclonal proliferation of mature B lymphocytes, typically leading to persistent lymphocytosis, peripheral lymphadenopathy, and bone marrow infiltration [5]. Extranodal involvement in CLL is rare, occurring in less than 5% of cases, most commonly affecting the skin, kidneys, lungs, and gastrointestinal tract [6]. Nasopharyngeal localization is exceptionally uncommon and has only been reported in isolated case reports, highlighting the clinical and diagnostic rarity of this presentation [1].

In our case, the diagnosis was established through a multimodal approach. Histology revealed a dense lymphoid proliferation composed of small, monomorphic lymphocytes, with an immunohistochemical profile consistent with CLL (CD20⁺, CD5⁺, CD23⁺, Bcl2⁺, cyclin D1⁻) and a Matutes score of 5/5. Cytogenetic analysis did not demonstrate a deletion of the p53 locus (17p13), a poor prognostic factor often associated with treatment resistance [7]. In addition to the absence of TP53 disruption, the presence of a mutated IGHV status in our patient represents a well-established favorable prognostic marker in CLL. IGHV-mutated CLL is typically associated with a more indolent disease course and improved responses to chemoimmunotherapy, which may partly explain the excellent clinical and radiological response achieved with rituximab-bendamustine in this case. Radiologically, CT and MRI confirmed a large nasopharyngeal mass filling the Rosenmüller fossae and Eustachian tube orifices, without evidence of bony involvement or locoregional spread, correlating well with the histopathological findings.

The pathophysiological mechanisms underlying extranodal involvement in CLL remain incompletely understood. Current evidence suggests that CLL tissue infiltration is not a random process, but rather the result of complex interactions between leukemic B cells and the tissue microenvironment. CLL cells exhibit altered expression of adhesion molecules and chemokine receptors, particularly CXCR4, CXCR5, and CCR7, which regulate homing to lymphoid and extranodal tissues through interactions with their ligands, such as CXCL12 and CXCL13 [8,9].

Furthermore, extranodal localizations in CLL have been associated with prolonged disease duration and specific biological profiles rather than with aggressive transformation in the absence of Richter syndrome [2]. In the present case, the absence of high-grade histological features and the low Ki-67 proliferation index argue against a transformation process and support the hypothesis of indolent extranodal infiltration driven by microenvironmental permissiveness rather than intrinsic tumor aggressiveness.

The favorable therapeutic response observed in this patient can be partly explained by the underlying molecular profile. The presence of a mutated IGHV status is a well-established prognostic marker in CLL and is consistently associated with a more indolent disease course, delayed disease progression, and improved overall survival [10]. IGHV-mutated CLL cells exhibit reduced B-cell receptor signaling capacity and lower proliferative drive, rendering them more sensitive to chemoimmunotherapy.

In parallel, the absence of TP53 disruption, as confirmed by the lack of 17p deletion on FISH analysis, represents a critical determinant of treatment response. TP53 alterations are strongly associated with genomic instability, chemoresistance, and inferior outcomes following conventional chemoimmunotherapy regimens [7,11]. In contrast, patients lacking TP53 abnormalities retain intact DNA damage response pathways, allowing effective apoptosis induction following alkylating agents such as bendamustine.

The coexistence of IGHV-mutated status and preserved TP53 function, therefore, constitutes a biologically favorable constellation, which has been shown to predict durable responses to rituximab-based chemoimmunotherapy [11]. This molecular background likely explains the complete clinical and radiological regression achieved in our patient, despite the unusual extranodal nasopharyngeal presentation. These findings support the notion that disease biology outweighs anatomical localization in determining therapeutic response in CLL.

In this case, the treatment consisted of six cycles of R-bendamustine, combining rituximab and bendamustine. This regimen is considered an effective option for CLL, particularly in patients under 65 years of age and without unfavorable cytogenetic abnormalities [12]. Several studies have demonstrated that R-bendamustine achieves high overall response rates while offering better hematologic tolerability than the FCR regimen (fludarabine, cyclophosphamide, rituximab) [13,14]. In our 64-year-old patient, the treatment was well tolerated and resulted in both clinical and radiological regression of the nasopharyngeal mass, along with improvement in obstructive symptoms.

This case highlights the importance of recognizing atypical presentations of CLL, particularly in unusual ENT locations. Such occurrences are rare and contribute significantly to the literature by enhancing clinical understanding of the diverse manifestations of CLL [1]. They also underscore the necessity of a rigorous histological and immunophenotypic diagnosis, preventing misdiagnosis as a primary epithelial malignancy and guiding an appropriate therapeutic strategy. Finally, this type of case emphasizes the value of a multidisciplinary follow-up, involving hematologists, radiologists, and ENT specialists, to optimize management and improve the overall prognosis for patients with atypical forms of CLL.

## Conclusions

Nasopharyngeal involvement in CLL is exceptionally rare and can mimic primary ENT tumors, posing a diagnostic challenge. Accurate diagnosis requires histological examination and immunophenotyping to distinguish CLL from primary nasopharyngeal malignancies. In this patient, R-bendamustine therapy led to complete regression of the nasopharyngeal lesion and normalization of hematologic parameters, consistent with the favorable prognosis associated with IGHV-mutated, TP53-intact CLL. This case underscores the importance of recognizing atypical extranodal presentations to guide timely and appropriate management.
